# Imbalance of Systemic Redox Biomarkers in Children with Epilepsy: Role of Ferroptosis

**DOI:** 10.3390/antiox10081267

**Published:** 2021-08-09

**Authors:** Sara Petrillo, Nicola Pietrafusa, Marina Trivisano, Costanza Calabrese, Francesca Saura, Maria Giovanna Gallo, Enrico Silvio Bertini, Federico Vigevano, Nicola Specchio, Fiorella Piemonte

**Affiliations:** 1 Unit of Muscular and Neurodegenerative Diseases, Bambino Gesù Children’s Hospital, IRCCS, Viale San Paolo 15, 00146 Rome, Italy; sara.petrillo@opbg.net (S.P.); mariagiovanna.gallo@opbg.net (M.G.G.); enricosilvio.bertini@opbg.net (E.S.B.); 2 Rare and Complex Epilepsy Unit, Department of Neurosciences, Bambino Gesù Children’s Hospital, IRCCS, Full Member of European Reference Network EpiCARE, Piazza S. Onofrio 4, 00165 Rome, Italy; nicola1.pietrafusa@opbg.net (N.P.); marina.trivisano@opbg.net (M.T.); costanza.calabrese@opbg.net (C.C.); 3 Department of Laboratory Medicine, Children’s Hospital Bambino Gesù, Piazza S. Onofrio 4, 00165 Rome, Italy; francesca.saura@opbg.net; 4 Department of Neuroscience, Bambino Gesu Children’s Hospital, IRCCS, Full Member of European Reference Network on Rare and Complex Epilepsies EpiCARE, Piazza S. Onforio 4, 00165 Rome, Italy; federico.vigevano@opbg.net (F.V.); nicola.specchio@opbg.net (N.S.)

**Keywords:** epilepsy, ferroptosis, NRF2, 4-HNE, GPX4

## Abstract

To assess if ferroptosis, a new type of programmed cell death accompanied by iron accumulation, lipid peroxidation, and glutathione depletion, occurs in children with epilepsy, and in order to identify a panel of biomarkers useful for patient stratification and innovative-targeted therapies, we measured ferroptosis biomarkers in blood from 83 unrelated children with a clinical diagnosis of epilepsy and 44 age-matched controls. We found a marked dysregulation of three ferroptosis key markers: a consistent increase of 4-hydroxy-2-nonenal (4-HNE), the main by-product of lipid peroxidation, a significant decrease of glutathione (GSH) levels, and a partial inactivation of the enzyme glutathione peroxidase 4 (GPX4), the mediator of lipid peroxides detoxification. Furthermore, we found a significant increase of NAPDH oxidase 2 (NOX2) in the blood of children, supporting this enzyme as a primary source of reactive oxygen species (ROS) in epilepsy. Additionally, since the nuclear factor erythroid 2-related factor 2 (NRF2) induction protects the brain from epileptic seizure damage, we also evaluated the NRF2 expression in the blood of children. The antioxidant and anti-inflammatory transcription factor was activated in patients, although not enough to re-establish a correct redox homeostasis for counteracting ferroptosis. Ferroptosis-mediated oxidative damage has been proposed as an emergent mechanism underlying the pathogenesis of epilepsy. Overall, our study confirms a crucial role for ferroptosis in epilepsy, leading to the identification of a panel of biomarkers useful to find new therapeutic targets. Developing innovative drugs, which act by inhibiting the ferroptosis signaling axis, may represent a promising strategy for new anti-seizure medications.

## 1. Introduction

Oxidative stress, glutamate-mediated excitotoxicity and neuroinflammation underlie the neurobiology of epilepsy, leading to seizure-induced cell death, increased susceptibility to neuronal synchronization, and progressive degeneration of brain areas [1,2,3,4]. Oxidative stress markers are increased in patients with status epilepticus (SE), correlating with severity, brain MRI, and outcome [1,2,5,6]. Moreover, targeting oxidative stress prevented epileptogenesis and protected against the cognitive patients decline [5].

Oxidative stress and antioxidant levels are important modulators of ferroptosis, which has been recently proposed as an emergent mechanism underlying the susceptibility to epileptic seizures [7,8]. Ferroptosis is an iron- and lipid-mediated programmed cell death characterized by ROS accumulation, GSH depletion, GPX4 decreased activity, and lipid peroxides accumulation [9]. In the epilepsy disease, ROS can act as primer, and ferroptosis underlies the excitotoxic neuronal injury in seizures [6,7].

Although several mechanisms trigger ferroptosis (GSH depletion, excess of glutamate, inhibition of GPX4), nevertheless many approaches (GSH augmentation, iron chelation, lipid radical scavenging) can revert it, thus paving the way for new promising therapeutic targets [10,11].

In this pilot cross-sectional study, we investigated if ferroptosis occurs in children with epilepsy by measuring the blood content of the “pathogenic triad” of ferroptosis bio-markers [8,12,13,14]; (i) 4-HNE and 15(S)-HETE, the main lipid peroxidation by-products [15,16]; (ii) GPX4, the principal lipid peroxides detoxifying enzyme [17]; and (iii) GSH, the primary ROS scavenger and cofactor for the GPX4 activity.

Furthermore, as the activation of the antioxidant transcription factor NRF2 can revert ferroptosis [9,10,11], and NRF2 appears promising as a neuroprotective factor in epileptogenesis and chronic epilepsy animal models [15,18,19,20,21,22], we evaluated the gene expression of NRF2 in the blood of children with epilepsy. Finally, in order to find the primary source of ROS in epilepsy, we also examined the leucocytes expression of NOX2, the enzyme responsible for the seizures-associated H_2_O_2_ release in epilepsy rodent models [15,18,19,20,21,22].

## 2. Materials and Methods

### 2.1. Participants’ Enrollment

A total of 83 unrelated patients (42 males, 41 females) and 44 age-matched healthy controls (23 males, 21 females) were enrolled within one year. All patients had a diagnosis of epilepsy and were consecutively seen in the outpatient clinic at the Department of Neuroscience, Bambino Gesù Children’s Hospital, IRCCS, Rome, Italy. Patients were classified based on epilepsy type, syndromes, and etiology following the current ILAE classification [23]. A patient was defined as drug resistant based on the ILAE updated definition [24]. The mean age of patients at epilepsy onset was 1.5 years (range 0–7 years), and the mean age at enrollment was 4 years (range 0.42–12 years). Twenty-nine patients out of 83 had a drug-resistant epilepsy. Patients with epilepsy symptomatic of brain tumors or neuro-degenerative disorders were excluded. The age, gender, and clinical phenotype of enrolled patients are presented in Table 1. The blood of age- and sex-matched healthy children, without history or clinical evidence of neurological, neuropsychological, oncological, and inflammatory diseases, was collected at the Department of Laboratory Medicine of the Bambino Gesù Children’s Hospital during routine blood tests. Because many conditions may affect the ferroptosis pathway [7], exclusion criteria, such as antioxidants supplementation, were adopted to prevent bias and confounding factors. All participants signed an informed consent, and the study was approved by the Ethics Committee of Bambino Gesù Children Hospital in Rome.

### 2.2. Blood Sample Collection

Blood samples were collected into EDTA Vacutainer Tubes (Becton Dickinson, Rutherford, NY) and leukocytes were isolated by adding 10% dextran. After 45 min at room temperature, the upper phase was centrifuged at 2600× *g* (5 min) and the pellet washed with 0.9% NaCl and stored at −20 °C until RNA and proteins extraction. Plasma was obtained by centrifuging whole blood at 450× *g* for 3 min and stored at −80 °C until 4-HNE and 15-(S)-HETE measurements.

### 2.3. Glutathione Assay

Glutathione levels were detected in whole blood by an enzymatic re-cycling assay. Samples were de-proteinized with 5% (*w*/*v*) sulphosalycilic acid (SSA, Sigma-Aldrich, St. Louis, MO, USA) and the glutathione content was determined after dilution of the acid-soluble fraction in Na-phosphate buffer containing EDTA (pH 7.5). GSH and GSH + GSSG concentrations were measured with the ThioStar^®^ glutathione detection reagent (Arbor Assays, Michigan, MI, USA), using GSH as standard (Sigma Chemicals, St. Louis, MO, USA), and expressed as µM. The fluorescence was measured by an EnSpire^®^ Multimode Plate Reader (Perkin Elmer, Waltham, MA, USA).

### 2.4. Determination of Plasma 4-HNE and 15(S)-HETE Content

Plasma 4-HNE and 15(S)-HETE concentrations were detected by competitive ELISA kits (Lipid Peroxidation 4-HNE and 15(S)-HETE Assay kits, Abcam, Cambridge, UK). Samples absorbance was detected on a microplate reader (Enspire, Perkin Elmer, Waltham, MA, USA) at 450 nm and quantified using a standard curve.

### 2.5. Quantitative Real-Time PCR (qRT-PCR)

Total RNA was extracted from leukocytes using Total RNA Purification Plus Kit (Norgen, Biotek Corp., Thorold, ON, Canada), according to manufacturer’s protocol. RNA quantification was performed on a NanoDrop2000 Spectrophotometer (Thermo Scientific, Waltham, MA, USA). The purity of RNA was assessed by measuring the ratio of absorbance at 260 nm and 280 nm. An amount of 1μg RNA was reverse transcribed with the SuperScriptTM First-Strand Synthesis system and random hexamers as primers (Life Technologies, Carlsbad, CA, USA). The expression levels of GCL, GPX4, and NRF2 were measured by qRT-PCR in an ABI PRISM 7500 Sequence Detection System (Life Technologies) using Power SYBR Green I dye chemistry (ThermoFisher Scientific, Walthman, MA, USA). Data were analyzed using the 2 ^∆∆^ Ct method with TBP (TATA box binding protein) as housekeeping gene and expressed as fold change relative to controls. Primers used for qRT-PCR are reported in Table 2.

### 2.6. Western Blot Analysis

Leukocytes were lysed on ice with RIPA buffer (Sigma Aldrich, St. Louis, Missouri, USA), including DTT and protease inhibitors (Protease and Phosphatase Inhibitor Mini Tablets, Thermo Scientific, Waltham, MA, USA). Proteins, in the amount of 40 μg, were subjected to SDS PAGE on 4–12% denaturing gel and probed with the following antibodies: Nrf2 (1:500, Abcam, Cambridge, UK), Nox2 (1:2000, Abcam, Cambridge, UK), and GAPDH (1:10,000, Sigma Aldrich) as loading control. Immuno-reactive bands were detected using the Lite Ablot Extend Long Lasting Chemiluminescent kit (Euroclone, Milan, Italy). HRP-conjugated secondary antibodies (Bethyl Laboratories, Montgomery, TX, USA) signals were captured by Chemi DocTM XRS 2015 (Bio-Rad Laboratories, Hercules, CA, USA). Densitometry was performed by Image Lab software (Version 5.2.1, Bio-Rad Laboratories). Proteins were quantified by Pierce™ BCA Protein Assay Kit (Thermo Scientific, Waltham, MA, USA).

### 2.7. GPX4 Enzyme Activity Assay

GPX4 activity was assessed in leukocytes by an enzyme-coupled test using the GPX4-specific substrate cumene hydroperoxide and the glutathione reductase catalyzed reduction of GSSG [25]. Leukocytes were resuspended in 100 μL lysis buffer (100 mM TrisHCL, pH 7.6, 5 mM EDTA, 1 mM NaN_3_, 0.1% Triton X-100) and 50 μg of protein extraction were added to a reaction mix consisting of 1 mL assay buffer (100 mM Tris, pH 7.8, 5 mM EDTA, 0.1% Triton X-100, 3 mM GSH, 0.2 mM nicotinamide adenine dinucleotide phosphate hydrogen (NADPH), 0.6 U/mL glutathione reductase, and 20 μM cumene hydroperoxide). The GPX4 activity was determined by measuring the decrease of NADPH absorbance at 340 nm. For normalization, protein concentration was measured using the BCA method (ThermoFisher, USA), according to the manufacturer’s instructions.

### 2.8. GST Enzyme Activity Assay

GST activity was determined in leukocytes by an enzyme-coupled assay using 1-Chloro-2,4-dinitrobenzene (CDNB) as specific substrate. GST assay was performed at 25 °C in 0.1 M phosphate-potassium buffer containing 0.1 mM EDTA. An amount of 25 µg of proteins was added to a reaction mix consisting of 1 mM GSH and 1 mM CDNB, and the increase of absorbance at 340 nm was followed on a microplate reader (Enspire, Perkin Elmer, USA). Data were normalized for protein concentration by the BCA method (ThermoFisher, USA), according to the manufacturer’s instructions.

### 2.9. Statistical Analysis

Estimated power analysis for two-sample comparison of means was assessed by Stata 14.1 (StataCorp LLC 4905, College Station, TX, USA). Statistical analysis was performed using the GRAPHPAD/Prism 5.0 Software (GraphPad Company, San Diego, CA, USA). Statistically significant differences between groups were analyzed using Student’s *t*-test for normally distributed variables. All experiments were performed in triplicates, and data are presented as mean ± standard error. Statistical significance was defined as * *p* < 0.05, ** *p* < 0.001, and *** *p* < 0.001 compared to healthy controls.

## 3. Results

### 3.1. Ferroptosis Bio-Markers Are Imbalanced in Children with Epilepsy

**Figure 1:**
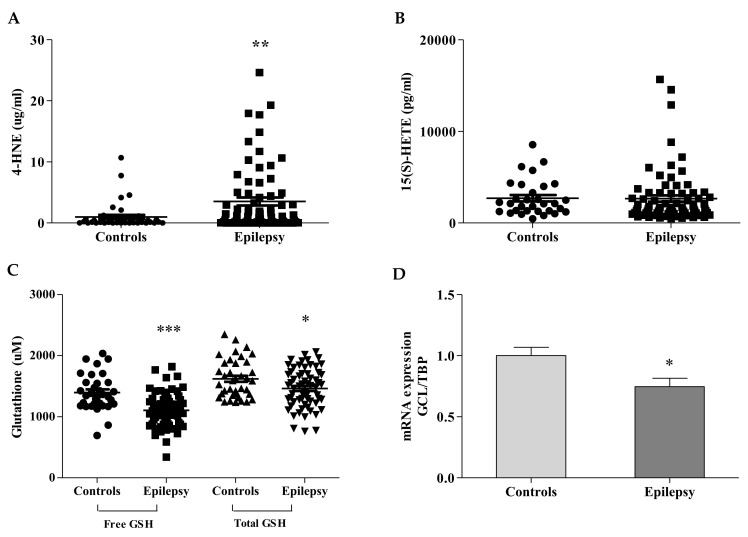
Ferroptosis biomarkers in blood of children with epilepsy. The lipid peroxidation products, 4-HNE (**A**) and 15 (S)-HETE (**B**), were measured in plasma of *n* = 83 patients and *n* = 44 controls (*n* = 31 controls for 15(S)-HETE); GSH concentration (**C**) and GCL gene expression (**D**) were detected, respectively, in blood and leukocytes, as reported in Materials and Methods. Values are expressed as median ± SEM. Statistical significance was defined as * *p* < 0.05, ** *p* < 0.01, *** *p* < 0.001 respect to controls.

Three ferroptosis markers were analyzed in blood of children with epilepsy: two by-products of lipid peroxidation (4-HNE, 15(S)-HETE) and GSH, the main antioxidant in cells, which displays dual actions, as a direct ROS scavenger and as co-factor in many detoxifying enzymes [4]. As shown in Figure 1A, the 4-HNE content was significantly increased in plasma of children with epilepsy (3.51 ± 0.64 µg/mL vs. 0.98 ± 0.32 healthy subjects, *p* < 0.01, 95% confidence interval = −4.252 to −0.8246), whereas the level of 15(S)-HETE, the principal metabolite produced by the arachidonate peroxidation, was comparable to controls (1967 ± 246 pg/mL vs. 2685 ± 350 pg/mL of healthy subjects, Figure 1B).

The glutathione concentration was significantly decreased in blood of patients, either in its free (not protein bound) form (1106 ± 35 µM vs. 1396 ± 52 µM healthy subjects, *p* < 0.0001, 95% confidence interval = 168.7 to 412.6), and in its total amount (1469 ± 41 µM vs. 1666 ± 53 healthy subjects, *p* < 0.01, 95% confidence interval = 27.94 to 284.9, Figure 1C). The qRT-PCR confirmed low expression levels of mRNA GCL (0.75 ± 0.07 vs. 1 ± 0.07 healthy subjects, Figure 1D), the gene coding for the step-limiting enzyme of the GSH synthesis.

**Figure 2:**
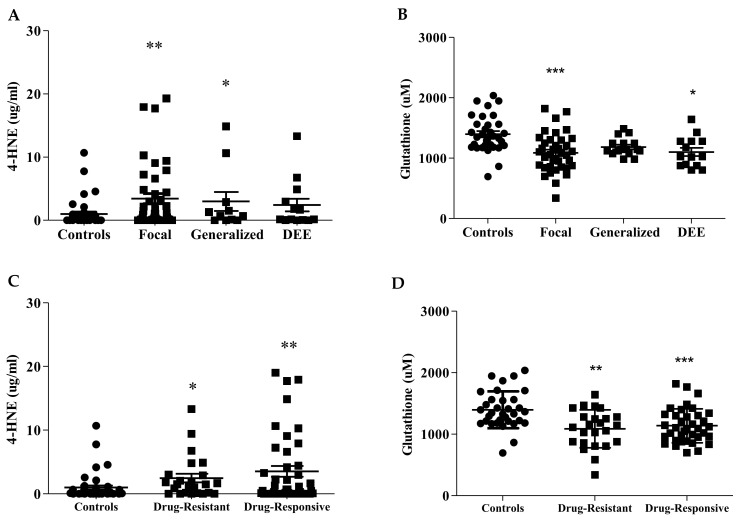
Ferroptosis biomarkers in sub-groups of children with epilepsy. The lipid peroxidation products, 4-HNE (**A**) and GSH (**B**), were determined in blood of children with focal epilepsy (*n* = 52), generalized epilepsy (*n* = 17), DEE (*n* = 14). 4-HNE (**C**) and GSH (**D**) were detected in blood of drug-resistant epilepsy (*n* = 29) and drug-responsive (*n* = 54) children. Data are expressed as median ± SEM. Statistical significance is defined as * *p* < 0.05, ** *p* < 0.01, *** *p* < 0.001 with respect to controls.

When we analyzed 4-HNE and GSH levels in patients’ sub-groups (Figure 2, Table 3), we had no significant differences either among patients with focal, generalized, or developmental and epileptic encephalopathy (DEE) epilepsy (Figure 2A,B), or between drug-resistant and drug-responsive patients (Figure 2C,D).

### 3.2. GPX4 Reflects the 4-HNE/GSH Blood Imbalance

**Figure 3:**
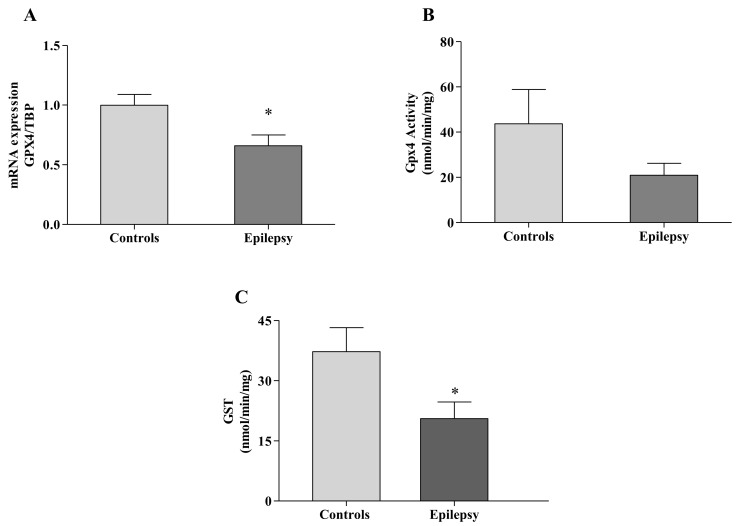
GPX4 and GST in blood of *n* = 83 children with epilepsy and *n* = 44 controls. Real-time PCR analysis (**A**) and enzymatic activity (**B**) of GPX4 in leukocytes of children with epilepsy. GST activity (**C**) was measured as reported in Methods. Enzyme activities are expressed as nmol/min/mg protein. Values are expressed as median ± SEM. Statistical significance is defined as * *p* < 0.05, respect to controls.

The overload of 4-HNE and the decrease of GSH represent a highly toxic combination in epilepsy because of their direct (by covalent binding) and indirect (by providing the reaction substrate) abilities to respectively inhibit GPX4 [12]. Therefore, we decided to analyze the GPX4 expression and activity in leukocytes of children with epilepsy. As reported in Figure 3A, the GPX4 transcript was significantly reduced in the disease (0.65 ± 0.09 vs. 1.00 ± 0.09 healthy subjects, *p* < 0.05), thus leading to a consistent decrease of the enzyme activity (21 ± 5 nmol/min/mg proteins vs. 44 ± 15 nmol/min/mg proteins in healthy subjects, Figure 3B).

### 3.3. GST Fails to Detoxify Ferroptosis By-Products in Epilepsy

Under physiological conditions, the 4-HNE overload is detoxified by GST, which catalyzes the conjugation of 4-HNE to GSH, removing its accumulation deleterious for cells [26]. Given that 4-HNE was high in blood of children with epilepsy and GSH, the co-factor of GST enzyme reaction, was significantly decreased, we measured the GST activity in our patients. As shown in Figure 3C, the GST activity was significantly reduced in leukocytes of children with epilepsy (20.5 ± 4 vs. 37.2 ± 6 nmol/min/mg healthy subjects, *p* < 0.05; Figure 3C), thus indicating a poor GST ability to buffer ferroptosis by-products in this disease.

### 3.4. NRF2 at the Crossroad of Redox Balance and Ferroptosis in Epilepsy

**Figure 4:**
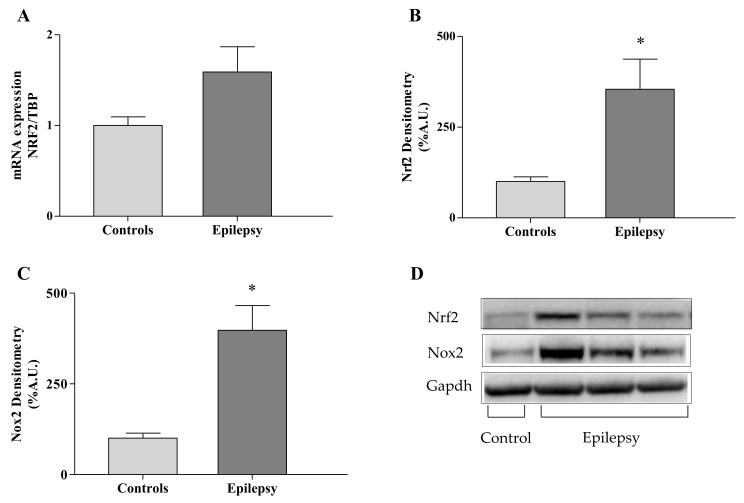
NRF2 and NOX2 expression in leukocytes of *n* = 83 children with epilepsy and in *n* = 44 controls. NRF2 mRNA (**A**) and protein amount (**B**,**D**), were determined by qRT-PCR and Western blot, respectively. NOX2 protein levels (**C**), as detected by Western blot analysis (**D**). Values are expressed as median ± SEM. Statistical significance is defined as * *p* < 0.05, respect to controls.

NRF2 is the main regulator of both GPX4 and GCL gene expression and is crucial in mediating the 4-HNE-induced response of antioxidant and detoxifying genes [18]. Thus, we asked if the upstream modulator NRF2 could be affected in epilepsy. As reported in Figure 4, we found an activation of NRF2 in patients, either as mRNA (1.53 ± 0.27 vs. 1.00 ± 0.09 healthy subjects, A) or as protein levels (354 ± 83 vs. 100 ± 13 healthy subjects, B), thus suggesting an attempt of tissues to counteract ferroptosis but not enough to re-establish a correct redox balance.

### 3.5. NOX2: A Primer of Ferroptosis in Epilepsy

Moving from previous studies showing that the activation of NOX2 is the primary trigger of epileptic seizures in rodent models and the major contributor to ROS production [27,28,29], we measured the expression of NOX2 in leukocytes of children with epilepsy. Consistent with previous studies, we found a significant increase of NOX2 expression (gp91-phox catalytic subunit) in our patients (397 ± 68 vs. 100 ± 14 healthy subjects, Figure 4C,D), supporting this enzyme as a key initiator of oxidative stress and ferroptosis in epilepsy.

## 4. Discussion

In this study, we tested the hypothesis that ferroptosis may occur in epilepsy, with the ultimate goal to identify a panel of predictive biomarkers that can be detected by blood analysis and potentially useful for patient stratification, individualized patient care, and innovative targeted therapies.

Peripheral blood is now considered a significant and easily accessible source of biomarkers for neurological diseases, able to mirror pathological changes occurring both at the central nervous system and cerebrospinal fluid levels [30,31]. To date, few human studies have examined candidate peripheral biomarkers in epilepsy [32]. Recently, Shekh-Ahmad [21] compared the total antioxidant capacity (TAC) in plasma, cortex, and hippocampus of an epilepsy mouse model, and they found decreased TAC levels in all districts, with comparable increases after antioxidant treatment, indicating that the biochemical changes occurring in SNC can be reflected in blood. Blood biomarkers as a surrogate for brain neurophysiology in epilepsy have also been analyzed by Liang et al. [33], who found elevated levels of blood S100B at seizure onset and after seizures in patients. Although more studies will be needed to demonstrate if defects in the CNS can be translated into blood biomarkers fluctuations, by this study, we explore the possibility that ferroptosis biomarkers can be an index of patient’s disease status and, potentially, progression.

Ferroptosis is a new type of cell death, usually accompanied by a large amount of iron accumulation and lipid peroxidation [9]. Direct and indirect evidence of neuronal ferroptosis have been proposed in seizure generation in several animal models of epilepsy [6] where, importantly, the inhibition of ferroptosis resulted to be neuro-protective [8]. A deeper understanding of ferroptosis in epilepsy may lead to clarify the pathogenic mechanism underlying seizure-induced neuronal death and contribute to reducing the deleterious impact of epileptic seizures on the brain, inducing cognitive, behavioral, and other neuropsychological disorders mostly in the developing brain. Furthermore, targeting ferroptosis and identifying biomarker profiles in patients may shed new light on the therapeutical approach in epilepsy, possibly modifying the neurobiological process beyond epilepsy.

The brain is particularly susceptible to ferroptosis, due to high oxygen consumption (approximately 20% of oxygen), low endogenous antioxidant defense, large quantity of iron and copper, and abundance of polyunsaturated fatty acids (PUFA) in neuronal membranes that are particularly prone to lipid peroxidation [34].

Lipid peroxidation affects membrane fluidity and permeability, producing many cytotoxic and reactive by-products. Lipid by-products have been found to increase in experimental models of epilepsy, further propagating oxidative damage [13,35,36,37,38]. In particular, lipid peroxides increased in the hippocampus of rat models during the acute phase of status epilepticus and persisted for several hours after spontaneous recovery, suggesting a wide therapeutic window for the use of antioxidants in the treatment of epilepsy [39,40].

However, despite the emerging role for ferroptosis in the epileptic phenotype, few studies have been performed in patients, most of them conducted in children receiving antiseizure medications [41,42,43]. An increased amount of lipid peroxides has been found in serum of children before treatment [44], but contrasting data have been reported in patients with newly diagnosed idiopathic epilepsy [45]. Thus, there is a growing search for biomarkers to allow more precise and earlier diagnoses, as well as improved prevention and treatment repurposing.

Our findings highlight a marked dysregulation of the three key markers of ferroptosis in blood of children with epilepsy. We found a consistent increase of 4-HNE, the main by-product of lipid peroxidation, a significant decrease of GSH levels, and a partial inactivation of the enzyme GPX4.

GPX4 is a crucial mediator of ferroptosis because of its role in detoxifying lipid peroxides and preventing their accumulation. The inhibition of GPX4 has been reported to induce early-onset seizures in mutant mice, with loss of hippocampal parvalbumin-positive (PV+) inhibitory interneurons and astrogliosis [46]. GPX4 gene mutations were also responsible for a rare pediatric syndrome (the Sedaghatian-type spondylometaphyseal dysplasia, OMIM #250220) characterized by severe neurological defects, seizures, and cerebellar hypoplasia [47].

The GPX4 uses GSH as a cofactor to reduce lipid peroxides to their corresponding alcohols, thereby limiting the formation of toxic radicals [17]. Therefore, GSH and GPX4 are closely related with each other, as well as 4-HNE that, when in excess, forms macromolecules-adducts, additionally contributing to GPX4 inactivation [9]. However, moderate increases of 4-HNE may act as a signaling molecule in cells, inducing the expression of the transcription factor NRF2 [15,18].

Thus, moving from the evidence that NRF2 activation protects the brain from damage induced by epileptic seizures [19,20,21], we additionally analyzed the expression of NRF2 gene in leukocytes of children with epilepsy.

Our findings show an activation of NRF2 signaling pathway in patients but, as proven by the persistently low levels of GSH and GPX4, is not sufficiently suitable in re-establishing the cellular redox homeostasis.

**Figure 5:**
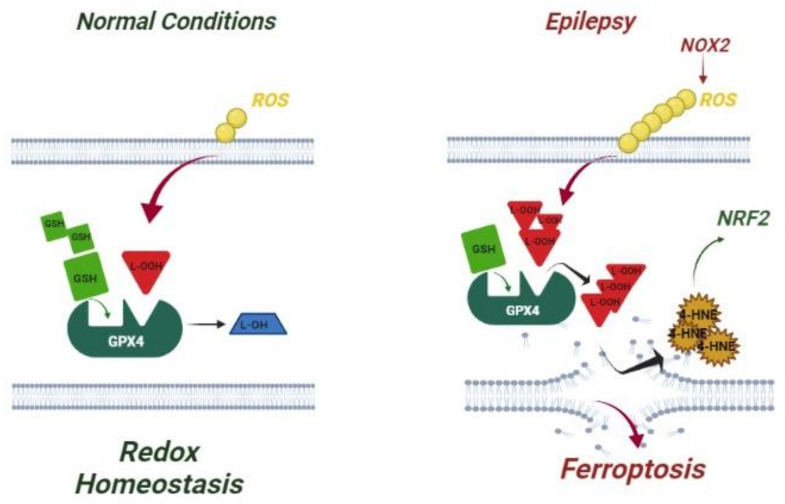
Representative model summarizing the main actors responsible for ferroptosis in epilepsy. In normal conditions, GPX4 detoxifies lipid hydroperoxides (L-OOH) by converting them to lipid alcohols (L-OH) and using GSH as a reaction cofactor. A balanced level of GSH and GPX4 ensures the maintenance of redox homeostasis. In epilepsy, the activation of NOX2 induces ROS overload with an increase of L-OOH that cannot be efficiently neutralized to L-OH because of decreased GSH and GPX4 contents. Consequently, L-OOHs accumulate, leading to ferroptosis and generation of L-OOHs by-products (4-HNE) that activate the antioxidant response (NRF2).

Then, to go deeper in the mechanism underlying ROS production in epilepsy and given the role of NOX as the main source of ROS production during seizure-like activity in animal models of epilepsy [13,28,48,49], we measured the expression of NOX in leukocytes of children with epilepsy. According to a recent study on a rodent model, in which the seizure onset was associated with a rapid release of NOX2-mediated H_2_O_2_ [29], we found a significant increase of protein amount in our patients, supporting NOX2 as the upstream trigger for the ferroptosis cascade in epilepsy (Figure 5).

Another enzyme has a key role in epilepsy, the GST, which belongs to the family of detoxifying enzymes, whose mechanism depends on the glutathione availability.

GST has been reported to contribute to drug-resistance in intractable patients with epilepsy [50], and it has been found highly expressed in the hippocampus of patients with Mesial temporal lobe epilepsy (MTLE), a form of epilepsy usually associated with drug-resistant seizures and cognitive deficits [51]. Moreover, Shang et al. [50] reported the activation of brain GST in drug-resistant patients, supporting this enzyme as responsible for seizures intractability. We found a significant decrease of GST activity in leukocytes of our patients, probably reflecting the low availability of its substrate GSH, which makes the enzyme inefficient in performing its detoxifying functions.

## 5. Conclusions

Collectively, our study highlights a role for ferroptosis in epilepsy, leading to identify a panel of predictive biomarkers potentially useful to discover early indication of disease and to find new targets for therapies in patients. Importantly, Zou et al. [37] demonstrated that the inhibition of ferroptosis suppressed epileptic seizures in experimental models and improved their cognitive deficits. This evidence, together with our data and the numerous encouraging results on the use of ascorbic acid [52], flavonoids [53] and vitamin E [43], in the prevention of epilepsy genesis, strengthen ferroptosis as a promising therapeutic approach in epilepsy. However, larger multicenter studies by recruiting children at the first episode of seizures and free of treatments will be needed to confirm and extend our observations and to evaluate the longitudinal evolution of biomarkers and their possible correlation with current therapies.

## Figures and Tables

**Table 1:** Demographic and clinical data of children with epilepsy and control group.

Variable	Controls	Patients
Age at enrollment, mean (range)	6 years (0.6–15)	4 years (0.42–12)
Age at epilepsy onset, mean (range)		1.5 years (0–7)
Sex, *n* (%)		
Male	23 (52%)	42 (51%)
Female	21 (48%)	41 (49%)
Drug-resistant, *n* (%)		29 (35%)
Drug-responsive, *n* (%)		54 (65%)
Focal epilepsy, *n* (%)		52 (63%)
Generalized epilepsy, *n* (%)		17 (20%)
DEE, *n* (%)		14 (17%)

Abbreviations. DEE: developmental and epileptic encephalopathy.

**Table 2:** Primers used for qRT-PCR analyses.

Genes	Sequence (5′ → 3′)	
GCL *601176	Fw-TTGCCTCCTGCTGTGTGATG	Rv-ATCATTGTGAGTCAACAGCTGTATGTC
GPX4 *138322	Fw-GCTCCATGCACGAGTTTTCC	Rv-ACTTCGGTCTTGCCTCACTG
NRF2 *600492	Fw-ACACGGTCCACAGCTCATC	Rv-TGTCAATCAAATCCATGTCCTG
TBP *605521	Fw-CCGAAACGCCGAATATAATCC	Rv-AAATCAGTGCCGTGGTTCGT

Abbreviations. GCL: Υ-glutamyl-cysteine ligase; GPX4: glutathione peroxidase 4; NRF2: nuclear factor erythroid 2-related factor 2; TBP: TATA box binding protein. * gene accession numbers

**Table 3:** Blood ferroptosis biomarkers in sub-groups of children with epilepsy.

Biomarkers	4-HNE, Mean ± SEM	Free GSH, Mean ± SEM	Total GSH, Mean ± SEM
Controls	0.98 ± 0.32	1396 ± 52	1621 ± 50
Focal epilepsy	3.44 ± 0.80 **	1088 ± 51 ***	1438 ± 55 *
Generalized epilepsy	2.97 ± 1.51 *	1235 ± 65	1492 ± 122
DEE	2.42 ± 1.00	1143 ± 84 *	1514 ± 135
Drug-resistant epilepsy	2.45 ± 0.68 *	1087 ± 75 **	1427 ± 87 *
Drug-responsive Epilepsy	3.52 ± 0.84 **	1135 ± 45 ***	1470 ± 54 *
Age at enrollment (months)			
0–12	5.64 ± 1.81 ***	1062 ± 74 **	1485 ± 107
12–24	3.235 ± 1.57 *	1161 ± 47 *	1472 ± 118
24–48	4.15 ± 1.40 **	1121 ± 71 **	1514 ± 81
48–144	2.52 ± 0.69	1117 ± 69 **	1436 ± 72 *
Epilepsy onset (months)			
0–12	3.66 ± 1.15 **	1034 ± 47 ***	1389 ± 89 *
12–24	4.15 ± 1.44 **	1188 ± 46 *	1545 ± 71
24–48	2.10 ± 0.73	1185 ± 99 *	1543 ± 92
48–144	2.58 ± 1.12	980 ± 113 ***	1273 ± 109 **

Abbreviations. 4-HNE: 4-Hydroxynonenal; DEE: developmental and epileptic encephalopathy. Data are reported as mean ± SEM (* *p* < 0.05, ** *p* < 0.01, *** *p* < 0.001, by Student’s *t*-test analysis).

## Data Availability

Data is contained within the article.
